# Bioinformatic features and immunological response of recombinant antigen CTLA4-IgV-EgG1Y162 against *Echinococcus granulosus*

**DOI:** 10.1590/1414-431X2024e13139

**Published:** 2024-11-25

**Authors:** Shangqi Zhao, Yanmin Li, Huifang Kong, Yanxia Zhou, Wentao Zhou, Jia Zheng, Qiaoqiao Gong, Chunbao Cao, Jianbing Ding, Xiaotao Zhou

**Affiliations:** 1 Department of Immunology, School of Basic Medical Sciences, Xinjiang Medical University, Urumqi, Xinjiang, China; 2 Xinjiang Key Laboratory of Molecular Biology for Endemic Diseases, Urumqi, Xinjiang, China; 3 The Fifth Affiliated Hospital of Xinjiang Medical University, Urumqi, Xinjiang, China

**Keywords:** Echinococcus granulosus, CTLA-4IgV-EgG1Y162, Bioinformatic analysis, DC, Immune response

## Abstract

Cystic echinococcosis (CE) is a zoonotic disease caused by the infection of *Echinococcus granulosus* (*E. granulosus*) larva. Currently, blocking the pathogenic cycle chain through immunoprophylaxis has become the main research direction. EgG1Y162 protein has good antigenicity and immunogenicity and is therefore a good candidate molecule for *E. granulosus* vaccine. Mature T cells express CTLA-4 on their surface, and its extracellular IgV region binds efficiently to the B7 molecules on antigen-presenting cells to deliver negative signals. We designed and prepared a recombinant vaccine by fusing CTLA-4IgV to the EgG1Y162 protein to exploit its binding properties. Bioinformatic methods were used to analyze the structure and epitopes of the proposed recombinant vaccine. The placement of 16 amino acids (GTDDDDKAMADIGSEF) between the CTLA-4IgV and EgG1Y162 using the skeleton structure of pET30a plasmid did not affect the correct folding of the proteins. When the recombinant proteins were co-cultured with bone marrow-induced dendritic cells (DC), the protein CTLA-4IgV-EgG1Y162 promoted its binding to DC and increased the percentage of DC maturation compared with protein EgG1Y162 *in vitro* and *in vivo*. Compared to EgG1Y162, CTLA-4IgV-EgG1Y162 promoted the proliferation of lymphocytes in spleen and the release of interferon (IFN)-γ and interleukin (IL)-4 by those lymphocytes *in vitro*, while it also promoted the release of protective antibodies in the serum of immunized mice *in vivo*. These findings indicated that the designed recombinant vaccine, CTLA-4IgV-EgG1Y162, can provide new ideas for the optimization and improvement of vaccines against *E. granulosus*.

## Introduction

Cystic echinococcosis (CE) is a zoonotic disease caused by the infection of *Echinococcus granulosus* metacestode (1). This disease commonly damages the liver, and the degree of damage is closely related to the site of parasitism, the size and number of cysts, and the nature and complications of the disease. No obvious signs or symptoms can be observed in the early stages. Over time, worm cysts grow in size and begin to compress the surrounding tissues and organs, which causes symptoms to appear. CE is often clinically diagnosed at an advanced stage (2). The conditions for the development of vaccines are gradually becoming more mature with the development of modern biological techniques; therefore, the search for and development of relevant protective vaccines against *E. granulosus* has become a current hot topic (3,4). A new gene has been described (5), *EgG1Y162*, in protoscoleces and adult worms of *E. granulosus* for the first time (GenBank accession No. AB462014), and the associated protein EgG1Y162 was later found to be a good vaccine candidate (6).

The extracellular IgV region of CTLA-4 can efficiently bind to the B7 molecules expressed on antigen-presenting cells (7). Animal immunization with a vaccine fused with CTLA-4IgV can produce a strong cellular and humoral immune response (8). Here, fusion protein CTLA-4IgV-EgG1Y162, as an improved hydatid vaccine, was expressed using the prokaryotic expression plasmid pET30a. We used the structure of plasmid pET30a to separate the two fused proteins by 16 amino acids (GTDDDDKAMADIGSEF), which was used to mimic the linker sequence to allow the two proteins to fold correctly by resisting interference with the spatial block. Bioinformatic methods (9) were used to predict the physicochemical properties, structure, and possible dominant antigenic epitopes of EgG1Y162 and CTLA-4IgV-EgG1Y162 proteins and to observe whether CTLA-4IgV and EgG1Y162 fused proteins could fold properly. Moreover, the fusion protein CTLA-4IgV-EgG1Y162 was tested to determine whether it was effective in enhancing the antigen-presenting ability of dendritic cells (DCs) and producing a stronger immune protection. This study provides a theoretical basis for the optimization and improvement of a hydatid fusion vaccine.

## Material and Methods

### Sequences of EgG1Y162 and CTLA-4IgV-EgG1Y162 proteins

The amino acid sequence of the EgG1Y162 protein (NCBI: AB458259) is VDPELMAKLTKELKTTLPEHFRWIHVGSRSLELGWNATGLANLHADHIKLTANLYTTYVTFKYRNVPIERQKLTLEGLKPSTFYEVVVQAFKGGSQVFKYTGFIRTLAPGEDGADRASGF (Supplementary Figure S1).

The amino acid sequence of the CTLA-4IgV-EgG1Y162 protein is NVTQPPVVLASSRGVASFTCEYESSGKADEVRVTVLRKAGIQVTEVCAGTYMVEDELTFLDDSSCIGTSRGNKVNLTIQGLRAMDTGLYVCKVELMYPPPYYMGEGNGTQIYVIDPEPCPDSDGTDDDDKAMADIGSEFVDPELMAKLTKELKTTLPEHFRWIHVGSRSLELGWNATGLANLHADHIKLTANLYTTYVTFKYRNVPIERQKLTLEGLKPSTFYEVVVQAFKGGSQVFKYTGFIRTLAPGEDGADRASGF (Supplementary Figure S2).

### Prediction software

The following prediction software programs were used: ProtParam (http://web.expasy.org/protparam/), TMHMM server (http://www.cbs.dtu.dk/services/TMHMM-2.0/), Self-optimized Prediction Method with Alignment (SOPMA) online analysis software (https://npsa-prabi.ibcp.fr/cgibin/npsa_automat.pl?page=npsa_sopma.html), Iterative Threading Assembly Refinement (I-TASSER (http://zhanglab.ccmb.med.umich.edu/I-TASSER/) (10,11), HDOCK, BepiPred 1.0 server (12) (http://www.cbs.dtu.dk/services/BepiPred-1.0/), Immune Epitope Database (IEDB; http://tools.iedb.org/main/bcell/, http://tools.iedb.org/MHC-II/), SVMTriP (http://sysbio.unl.edu/SVMTriP/prediction.php), and SYFPEITHI (http://www.syfpeithi.de/bin/mhcserver.dll/epitopeprediction).

### Experimental animals

Specific pathogen-free C57 mice (6-8 weeks, 20±2 g) and BALB/C mice were purchased from the Experimental Animal Center of Xinjiang Medical University, China (License No. SCXK(Xin)2016-0003).

### Bioinformatic analysis of EgG1Y162 and CTLA-4IgV-EgG1Y162 proteins

The physicochemical properties of EgG1Y162 and CTLA-4IgV-EgG1Y162 proteins were analyzed using the online software ProtParam. The TMHMM server was used to analyze the transmembrane domains of the recombinant proteins. SOPMA and I-TASSER were used to predict the secondary and tertiary structures of the proteins, respectively. BepiPred, IEDB, and other software programs were used to predict proteins and T/B antigenic epitopes. All protein structures were processed on the platform, including removing water and ions, protonation, adding lost atoms and missing groups, and minimizing the energy between the proteins. The docking between the EgG1Y162/CTLA-4IgV-EgG1Y162 and CD80 expressed on the surface of the antigen presenting cells was carried out by using the HDOCK software. The docking model was modified by the software MOE, and the structure of the blocking protein was analyzed by PyMOL (https://www.pymol.org/).

### Expression, identification, and specificity of the EgG1Y162 and CTLA-4IgV-EgG1Y162 proteins

Recombinant plasmids pET30a-EgG1Y162 and pET30a-CTLA-4IgV-EgG1Y162 were transformed into *Escherichia coli*BL21 (DE3) cells. The bacterial culture containing the recombinant plasmid pET30a-EgG1Y162 was induced at 28°C with 0.2 mmol/L IPTG (Solarbio, China) for 6 h. The strain solution containing the plasmid pET30a-CTLA-4IgV-EgG1Y162 was induced at 28°C with 0.2 mmol/L IPTG for 2 h and then transferred to 37°C for 2 h. The thalli were crushed by ultrasound, and the supernatant was collected through a His-Trap column (General Electric, USA). The EgG1Y162 and CTLA-4IgV-EgG1Y162 proteins were eluted with pre-prepared imidazole (13). The purified proteins were collected, and ultrafiltration was performed using a concentration column (GE Healthcare, USA). The target proteins were identified by western blot (14). His-Tag antibody (Cell Signaling Techonology, USA) was used for western blot detection. The specificity of the protein was tested by the antibody in the serum of CE-infected mice and healthy mice and in the serum of a CE patient and a healthy person by western blot. Goat anti-mouse IgG antibody (HRP) and goat anti-human IgG antibody (HRP) were used as secondary antibodies (Aibixin Shanghai Biotechnology Co., LTD.,China).

### Binding ability of DCs to the protein *in vitro*

The femur and tibia of mice were removed under aseptic conditions after the C57 mice were sacrificed by cervical dislocation. Bone marrow hematopoietic stem cells were collected, made into a cell suspension (3×10^6^ cells/mL), and placed onto a six-well plate with 3 mL for each well. rmGM-CSF (final concentration of 20 ng/mL) and rmIL-4 (final concentration of 10 ng/mL) (Peprotech, USA) were added and placed in a cell culture incubator (Thermo, USA) to obtain DCs (15). The induced DCs were randomly divided into groups A and B, which were stimulated with 500 ng/mL His-EgG1Y162 and His-CTLA-4IgV-EgG1Y162, respectively, for 24 h. Approximately 1×10^6^ cells were placed in each flow tube, and PBS was added to each tube. The cells were mixed and centrifuged at 100 *g* and 4°C for 5 min. The supernatant was discarded, and anti-His Tag antibody (Cell Signaling Technology, USA) was added and incubated. The expression of antigen molecules bound to DC surface was detected by flow cytometry (BD Biosciences, USA) within 4 h.

### Maturation of DCs stimulated by the protein *in vitro* and *in vivo*

DCs were collected after incubation with two different antigens (His-EgG1Y162 and His-CTLA-4IgV-EgG1Y162) for 24 h *in vitro*. DCs were collected from the spleen of immunized mice *in vivo*. Approximately 1×10^6^ cells were placed in each flow tube, and PBS was added to each tube. The cells were centrifuged at 100 *g* and 4°C for 5 min. The supernatant was discarded, and CD86^+^ antibody, CD11C^+^ antibody, CD45^+^ antibody, and I-Ab antibody (BD Biosciences) were added and incubated at 4°C for 30 min (16). The percentage of mature dendritic cells (mDCs) was detected by flow cytometry within 4 h.

### Function of spleen lymphocytes in the immunized mouse

Thirty-six female BALB/C mice were randomly divided into four groups (six mice per group): the control group, the negative group injected with the adjuvant (Sigma, USA), the EgG1Y162 test group, and the CTLA-4IgV-EgG1Y162 test group. All mice were immunized three times on days 1, 14, and 28. To the EgG1Y162 test group and CTLA-4IgV-EgG1Y162 test group, 50 μg recombinant protein was injected; 50 μg adjuvant was injected 3 times in the negative group, while mice in the control group were normally fed without any injection. The spleen cell suspension from the immunized mice was prepared with RPMI-1640 containing 10% FBS, and the cell concentration was adjusted to 5×10^6^/mL. We cultured 300 μL of cell suspension in a well of a 48-well plate for 48 h. Recombinant proteins (1.5 mg) were added to spleen cells of the EgG1Y162 test group and the CTLA-4IgV-EgG1Y162 test group, while RPMI-1640 culture medium was added to cells of the negative group; no stimulus was added to cells of the control group. After 48 h, the proliferation of spleen cells was detected by CCK8 test kits (TransGen Biotech, China) (17), and the cell supernatants of each group were for the detection of the concentration of interleukin (IL)-4 and interferon (IFN)-γ using ELISA kits (MultiSciences, China) (18).

### Titer of specific antibodies (IgG, IgG1, IgG2a) by ELISA

The titers of antibodies were detected in each group by ELISA (19). We coated 300 ng EgG1Y162 to a well in a 96-well plate at 4°C overnight. After being washed with 1 ×PBST, the plate was blocked by non-fat milk/PBST at 37°C for 2 h. The blocked plate was washed with 1 ×PBST and then 50 μL serum was added to each group at different dilutions (1:300, 1:900, 1:2700, 1:8100, 1:24,300, 1:72,900, 1:218,700, 1:656,100) in every well and cultured at 37°C for 1 h. After being washed 3 times, 50 μL of 1:50,000 goat anti-mouse IgG-HRP, goat anti-mouse IgG1-HRP, and goat anti-mouse IgG2a-HRP were added to each well and cultured at 37°C for 40 min. HRP was activated by TMB (Xinsaimei Biotechnology Co., Ltd., China), and the reaction was stopped by 2 mM H_2_SO_4_. The absorbance value was immediately detected at 450 nm.

### Statistical analysis

All data are reported as means±SD, and statistical analysis was done using SPSS 22.0 software (IBM, USA). Data of multiple groups were analyzed by one-way analysis of variance. A P value less than 0.05 indicated significant difference. GraphPad Prism 5.0 (USA) was used to draw the map.

## Results

### Bioinformatic analysis predicted that EgG1Y162 and CTLA-4IgV-EgG1Y162 proteins could fold normally

#### Physicochemical properties of the recombinant proteins

The EgG1Y162 protein has 120 amino acids, a protein molecular weight of 13,515.49 Da, a theoretical isoelectric point (PI) value of 9.22, a chemical formula of C_619_H_960_N_164_O_174_S_1_, an extinction coefficient of 18,450, an instability index of 29.98 (instability coefficient <40 indicates instability), and a grand average of hydropathy (GRAVY) score of −0.263 (the overall GRAVY range is between −2 and 2, and negative numbers indicate hydrophilicity).

The CTLA-4IgV-EgG1Y162 protein has 259 amino acids, a protein molecular weight of 28,491.22 Da, a theoretical PI value of 4.94, a chemical formula of C_1265_H_1973_N_331_O_395_S_11_, an extinction coefficient of 29,130, an instability index of 33.76, and a GRAVY score of −0.247.

**Figure 1:**
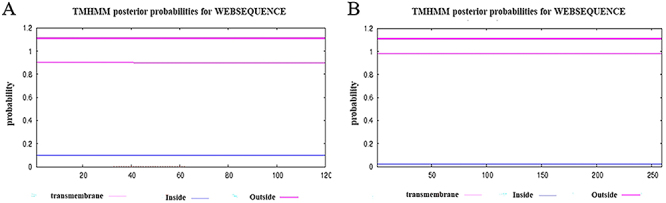
Lack of transmembrane domain of the recombinant proteins. **A**, EgG1Y162. **B**, CTLA-4IgV-EgG1Y162.

The TMHMM server was used to analyze the transmembrane regions of EgG1Y162 and CTLA-4IgV-EgG1Y162 (Figure 1A and B). The transmembrane regions of the two proteins were greater than 1, which suggested that EgG1Y162 and CTLA-4IgV-EgG1Y162 are soluble proteins that can be completely contacted by antigen-presenting cells and induce T and B cells to produce a strong immune response.

#### Prediction of the secondary structures of the recombinant proteins

**Figure 2:**
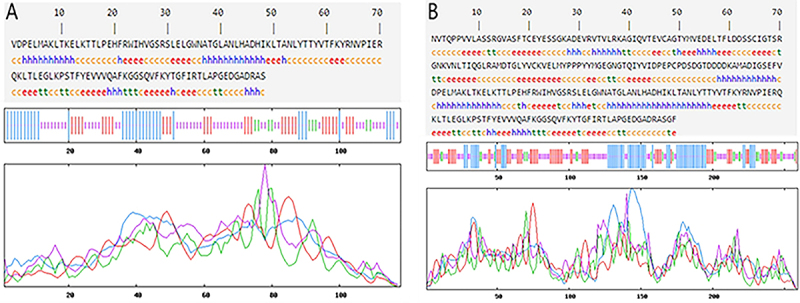
Secondary structure of the recombinant proteins was analyzed by SOPMA (https://npsa-prabi.ibcp.fr/cgibin/npsa_automat.pl?page=npsa_sopma.html). **A**, EgG1Y162. **B**, CTLA-4IgV-EgG1Y162.

The secondary structures of the recombinant proteins were analyzed by SOPMA software. The composition of the secondary structure of EgG1Y162 is shown in Figure 2A, where the α-helix, β-fold, β-turn, and random coils were 22.50, 29.17, 5.00, and 43.33%, respectively. The composition of the secondary structure of CTLA-4IgV-EgG1Y162 is shown in Figure 2B, where the α-helix, β-fold, β-turn, and random coils were 27.03, 27.80, 10.04, and 35.14%, respectively. According to the characteristics of spatial conformation, β-turn and random coils are mostly located on the surface of proteins; their location is beneficial to binding with antibodies; therefore, these structures are more likely to become epitopes (20).

#### Prediction of the tertiary structure and epitopes of the recombinant proteins

**Figure 3:**
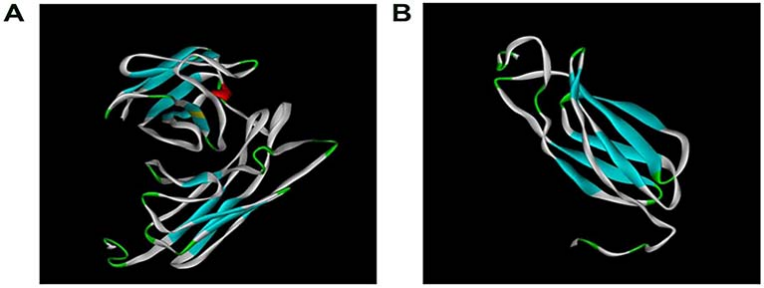
Tertiary structure of the recombinant proteins. **A**, CTLA-4IgV-EgG1Y162, constituted by two parts, CTLA-4 IgV and EgG1Y162. **B**, EgG1Y162.

I-TASSER was used to predict the tertiary structures of proteins (10,21). In the prediction of the tertiary structure of the recombinant protein CTLA-4IgV-EgG1Y162 (Figure 3A), the C-score was −1.69 (C-score usually ranges between −5 and 2, with a higher score indicating higher credibility), TM-score was 0.51±0.15 (TM-score >0.5 shows a correct topology model, whereas TM-score <0.17 often prompts a random similarity model), and root-mean-square deviation (RMSD) was 9.7±4.6 Å. In the prediction of the tertiary structure of EgG1Y162 (Figure 3B), the C-score was −1.55, TM-score was 0.52±0.15, and RMSD was 7.6±4.3 Å.

The possible dominant epitopes of recombinant proteins on T/B cells were predicted by BepiPred 1.0, SVMTriP, IEBD, and SYFPEITHI software. The predicted results showed that EgG1Y162 had three B-antigen epitopes with 19-28, 60-75, and 108-117 amino acids and five T epitopes with 7-18, 22-32, 47-71, 77-91, and 95-118 amino acids. CTLA-4IgV-EgG1Y162 has three B-antigen epitopes with 158-167, 199-209, and 247-256 amino acids and five T-epitopes with 146-160, 163-175, 184-208, 216-228, and 234-250 amino acids. The obtained T/B epitopes of EgG1Y162 and CTLA-4IgV-EgG1Y162 were similar, which means that the T/B epitope of the recombinant vaccine CTLA-4IgV-EgG1Y162 is also effective (Supplementary Table S1).

#### Interaction between CTLA-4IgV-EgG1Y162/EgG1Y162 and B7

**Figure 4:**
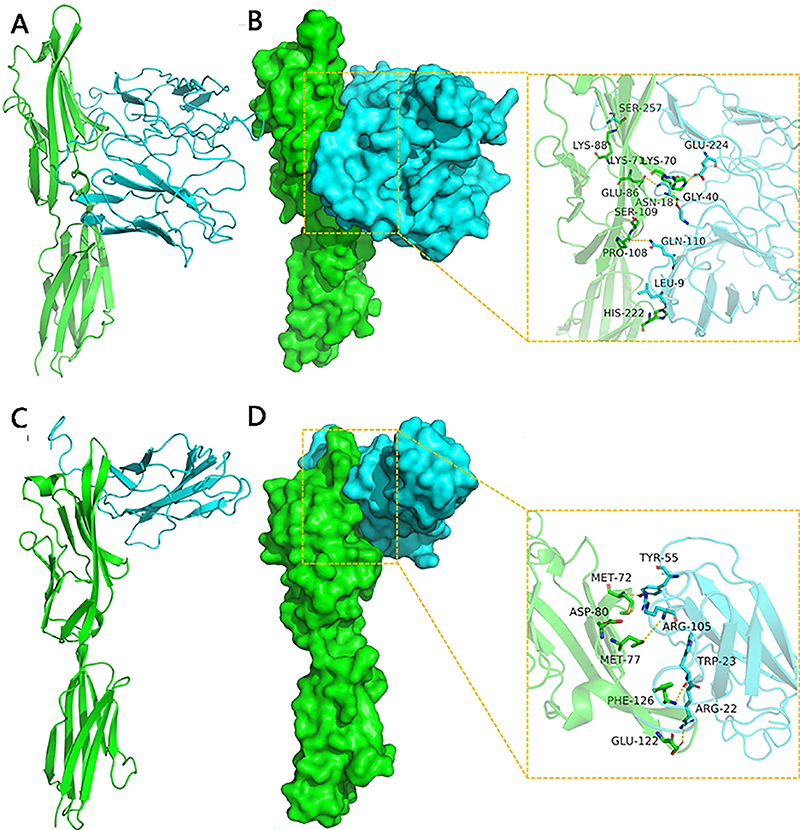
Docking between the recombinant proteins and B7. **A**, The backbones of B7 and CTLA-4IgV-EgG1Y162 proteins are shown in green and blue, respectively. **B**, Binding surface between B7 (green) and CTLA-4IgV-EgG1Y162 (blue); the hydrogen bond or salt bridge is shown in the yellow frame. **C**, The backbones of B7 and EgG1Y162 proteins are shown in green and blue, respectively. **D**, Binding surface between B7 (green) and EgG1Y162 (blue); the hydrogen bond or salt bridge is shown in the yellow frame.

The docking between CTLA-4IgV-EgG1Y162/EgG1Y162 and B7 on antigen-presenting cells was analyzed by using HDOCK, and the whole surface was set as the contact site. One hundred conformations were obtained after docking, and the conformation with the most negative energy was selected. The result showed that the score of the binding between CTLA-4IgV-EgG1Y162 and B7 was -282.14 kcal/mol, and the binding sites to B7 included Lys-71, Lys-70, his-222, pro-180, Glu-86, and Lys-88 of CTLA-4IgV-EgG1Y162. The binding sites to CTLA-4IgV-EgG1Y162 included Glu-224, Gly-40, Leu-9, Gln-110, ASN-181, and Ser-257 of B7. Meanwhile, the score of the binding between EgG1Y162 and B7 was -226.57 kcal/mol. The binding sites to B7 protein included Phe-126, Glu-122, Met-72, Met-77, and ASP-80 of EgG1Y162. The binding sites to EgG1Y162 included Trp-23, Arg-22, and Tyr of B7 (Figure 4).

### HIS-EgG1Y162 and HIS-CTLA-4IgV-EgG1Y162 recombinant proteins were expressed correctly and both had perfect specificity

**Figure 5:**
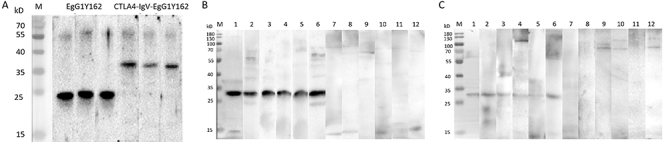
Western blot detection of the recombinant proteins in sera from mice and humans. **A**, Identification of the recombinant proteins. M: protein molecular quality standard; 1-3: HIS-EgG1Y162 recombinant protein; 4-6: HIS-CTLA-4IgV-EgG1Y162 recombinant protein. **B**, Detection in mouse serum. M: protein molecular quality standard; 1-6: cystic echinococcosis (CE) infected mice; 7-12: healthy mice. **C**, Detection in human serum. M: protein molecular quality standard; 1-6: CE patient; 7-12: healthy subject.

The recombinant proteins HIS-EgG1Y162 and HIS-CTLA-4IgV-EgG1Y162 were purified under optimal conditions and showed clear bands at 20.5 and 34.3 kDa, respectively, which were in accordance with the expected results (Figure 5A). When it was tested in the serum of CE-infected mice and healthy mice and in a CE-infected patient and a healthy person, the specificity of CTLA-4IgV-EgG1Y162 was 100% (Figure 5B and C).

### CTLA-4IgV promoted the binding of EgG1Y162 to DCs *in vitro*

**Figure 6:**
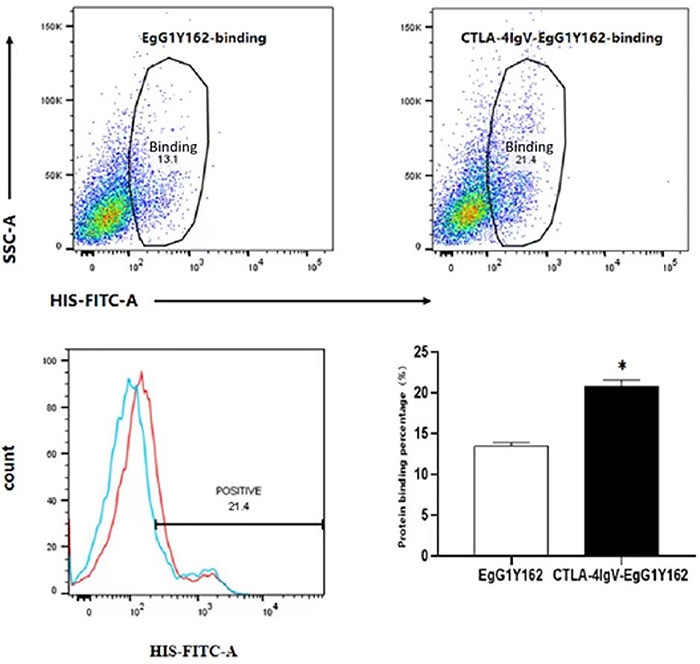
Binding between HIS-EgG1Y162 and HIS-CTLA-4IgV-EgG1Y162 with B7 expressed on dendritic cells (DCs). Compared to HIS-EgG1Y162, the insertion of the N-terminal CTLA-4IgV promoted the binding of EgG1Y162 to DCs. Data are reported as means and SD. *P<0.05, compared to HIS-EgG1Y162 group (Student's *t*-test).

After 24 h of co-culture with each of the two proteins, the his-tag antibody used in the binding assay was conjugated with FITC, and the result showed that the percentage of DCs that combined with HIS-CTLA-4IgV-EgG1Y162 (20.9±0.7%) was significantly higher than those that combined with HIS-EgG1Y162 (13.5±0.458%) (t=-15.379, P<0.01) (Figure 6).

### CTLA-4IgV-EgG1Y162 promoted DC maturation *in vitro* and *in vivo*

**Figure 7:**
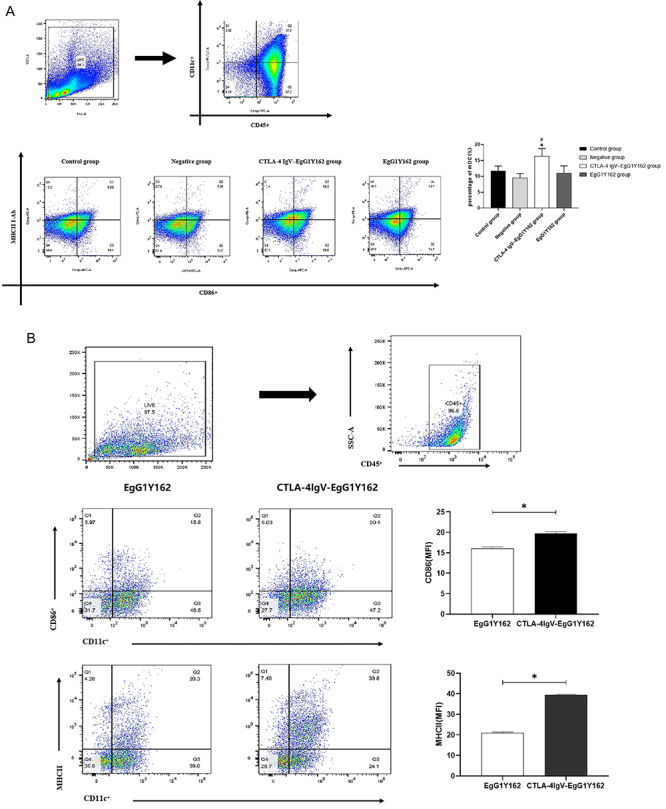
Flow cytometry was used to detect mature dendritic cells (mDCs). **A**, The maturity of DCs after it was cultured with HIS-EgG1Y162/HIS-CTLA-4IgV-EgG1Y162 for 24 h. *P<0.05, compared to control and negative groups; ^#^P<0.05, compared to EgG1Y162 group. **B**, The mDC of spleen of mice in each group. Data are reported as means and SD. *P<0.05, compared to EgG1Y162 group (Student's *t*-test).

The mDC in the spleen of each group was detected by flow cytometry, and the results showed that the percentages of CD11c+CD86+MHC-II+ phenotypes of CD45-positive immune cells were 16.360±2.414% and 11.010±2.299% in the CTLA-4IgV-EgG1Y162 group and in the EgG1Y162 group, respectively. In addition, the percentages of CD11c+CD86+MHC-II+ phenotypes in CD45-positive immune cells were 11.720±1.539% and 9.436±1.472% in the control group and in the negative group, respectively. Compared to those of the control group, the negative group, and the EgG1Y162 group, the percentage of mDC in the CTLA-4IgV-EgG1Y162 group was significantly increased (P<0.05) (Figure 7A and Supplementary Table S2). Flow cytometry was used to detect the expression of DC surface marker (CD11c) and mDC surface markers (MHC-II and CD86) in CD45-positive immune cells after antigen stimulation for 24 h *in vitro*. Figure 7B shows that after 24 h of EgG1Y162 antigen stimulation, the percentages of CD11c+CD86+ and CD11c+MHC-II+ phenotypes in CD45-positive immune cells were 16.033±0.404% and 20.8 ±0.625%, respectively. After 24 h of His-CTLA-4IgV-EgG1Y162 antigen stimulation, the percentages of CD11c+CD86+ and CD11c+MHC-II+ phenotypic immune cells in CD45-positive immune cells were 19.733±0.404% and 39.433±0.351%, respectively. The percentage of mDC significantly increased, and the difference was statistically significant (t=11.213, P<0.01; t=45.046, P<0.01).

### CTLA-4IgV-EgG1Y162 promoted the proliferation of lymphocytes in spleen and IL-4 and IFN-γ secretion

**Figure 8:**
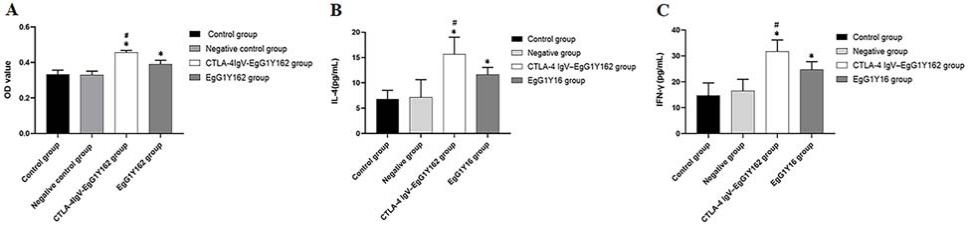
**A**, Analysis of splenic lymphocyte proliferation, (**B**) interleukin (IL)-4 expression level, and (**C**) interferon (IFN)-γ expression level in each group. Data are reported as means and SD. *P<0.05 compared to control group and negative group; ^#^P<0.05 compared to EgG1Y162 group (ANOVA).

The proliferation of lymphocytes in spleen stimulated by CTLA-4IgV-EgG1Y162 and EgG1Y162 increased significantly compared to the control group and the negative group (P<0.05). Compared to EgG1Y162, lymphocytes in spleen stimulated by CTLA-4 IgV-EgG1Y162 had a stronger proliferation ability (P<0.05) (Figure 8A, Supplementary Table S3). After spleen lymphocytes were stimulated with the proteins, the level of IL-4 (Figure 8B,) and IFN-γ (Figure 8C,) secreted by the lymphocytes in the spleen was increased in the CTLA-4IgV-EgG1Y162 group and the EgG1Y162 group (P<0.05). Furthermore, the level of IL-4 and IFN-γ in the CTLA-4IgV-EgG1Y162 group was higher than those in the EgG1Y16 group after stimulation (P<0.05) (Supplementary Table S4).

### CTLA-4IgV-EgG1Y162 enhanced the secretion of a series of antibody subtypes

**Figure 9:**
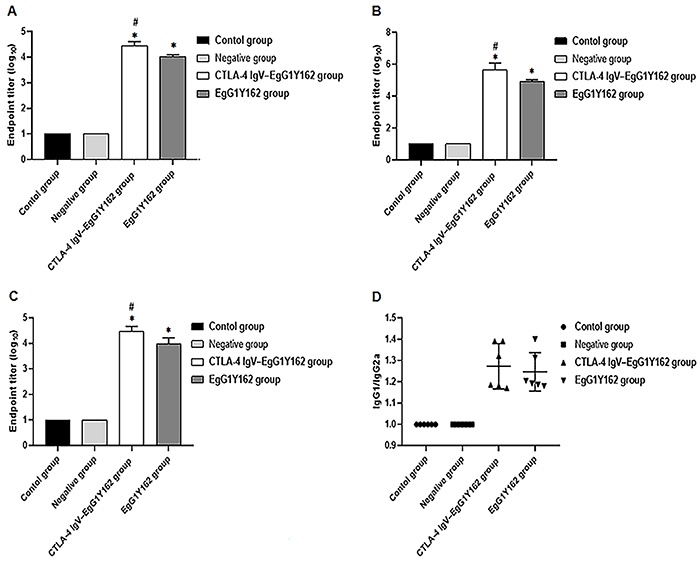
Antibody titers in peripheral blood of mice were detected by ELISA. **A**, Specific antibody IgG in mouse serum; **B**, Specific antibody subtype IgG1 in mouse serum; **C**, Specific antibody subtype IgG2a in mouse serum; **D**, Specific antibody subtype IgG1/IgG2a in mouse serum. Data are reported as means and SD. *P<0.05 compared to control group and negative group; ^#^P<0.05 compared to EgG1Y162 group (ANOVA).

After three immunizations, mice were sacrificed and serum was collected. The level of antigen-specific protective antibody IgG and its subtypes (IgG1, IgG2a) were detected by ELISA. The results showed that the level of IgG in the CTLA-4IgV-EgG1Y162 group was 10^4.457±0.153^, and the level of IgG in the EgG1Y162 group was 10^4.033±0.063^, which were higher than those of the negative control group and the normal control group (P<0.05) (Figure 9A). In addtion, compared to EgG1Y162, IgG production was increased in serum of mice immunized with CTLA-4IgV-EgG1Y162 (P<0.05). The levels of IgG1 and IgG2a in the CTLA-4IgV-EgG1Y162 group were 10^5.665±0.425^ and 10^4.454±0.209^, respectively. The levels of IgG1 and IgG2a in the EgG1Y162 group were 10^4.926±0.108^ and 10^3.966±0.253^, respectively (Figure 9B and C). The titer of IgG1 and IgG2a was the same as those of IgG, which proved that CTLA-4IgV-EgG1Y162 could induce a strong immune response and showed a protective effect to the body. Also, the value of IgG1/IgG2a in the serum of immunized mice was higher than 1 (Figure 9D).

## Discussion

CE is a zoonotic disease that affects the liver and lungs of humans and other animals caused by parasitism (22). When the disease compresses surrounding tissues, tissue atrophy and dysfunction occurs. After cyst rupture, the disease can also cause an allergic reaction and can be even life-threatening in serious cases (23). Current CE treatment relies on surgery and drugs, but the therapeutic effect is not ideal. The Eg95 vaccine for hydatid disease is well researched, but its reliability and practicability are still limited (24). Therefore, the screening and improvement of candidate vaccines have been the focus of research. EgG1Y162 was cloned from *E. granulosus* protoscolices and adult *E. granulosus* by PCR amplification (5).

EgG1Y162 has good antigenicity and immunogenicity and is therefore a good candidate molecule for *E. granulosus* vaccine (6). *E. granulosus* larvae can infect intermediate hosts such as sheep, and EgG1Y162 is an important antigen gene in this stage. CTLA-4 (CD152) is a ligand protein molecule on the surface of active T lymphocytes involved in immune inhibitory signaling transduction (25). Its monomeric molecule is composed of characteristic IgV extracellular, transmembrane, and intracellular regions (7). The extracellular region of CTLA-4 is unable to conduct negative regulatory signals because of the absence of transmembrane and intracellular regions, but its efficient binding function to B7 molecules on the surface of antigen-presenting cells is still present. The IgV region of CTLA-4 is very effective in enhancing the immune response of DNA vaccine, particularly Th1 response and CTL response activities after the animals being immunized with a DNA vaccine conjugated to CTLA-4 IgV (26). Therefore, we fused the extracellular IgV region of CTLA-4 to the EgG1Y162 antigen to take advantage of the fact that the extracellular region of CTLA-4 binds efficiently to the B7 molecule on antigen-presenting cells; thus, we hypothesized that the antigen could bind efficiently to antigen-presenting cells and produce a strong immune response.

The correct folding of proteins requires a certain amount of space. Two closely adjacent proteins often interfere with each other and cannot fold correctly because of steric hindrance (27). In the constructed plasmid, pET30a-CTLA-4IgV-EgG1Y162, CTLA-4IgV, and HIS-EgG1Y162 are separated by 16 amino acids (GTDDDDKAMADIGSEF) using the original backbone of the plasmid, which separates CTLA-4IgV from EgG1Y162 and facilitates the normal folding of adjacent proteins. In this study, the structures of EgG1Y162 and CTLA-4IgV-EgG1Y162 were predicted separately by bioinformatic methods. The addition of CTLA-4IgV at the N-terminal of EgG1Y162 did not change its physicochemical properties, hydrophilicity, or stability. If the antigenic epitope of protein EgG1Y162 is unchanged in the CTLA-4IgV-EgG1Y162 protein, then EgG1Y162 can perform its original role in the recombinant protein. Thus, we performed bioinformatic predictions of the antigenic epitopes of the recombinant proteins. We found by data integration that the T/B epitopes predicted for CTLA-4IgV-EgG1Y162 are distributed at positions 146-167 (on positions 7-28 of EgG1Y162, “AKLTKELKTTLPEHFRWIHVGS”), 184-209 (on positions 45-70 of EgG1Y162, “ADHIKLTANLYTTYVTFKYRNVPIER”), and 234-256 (on positions 95-117 of EgG1Y162, “SQVLAPGEDGDR”). The T/B antigen epitopes for EgG1Y162 protein are distributed on positions 7-28 (“AKLTKELKTTLPEHFRWIHVGS”), 47-75 (“HIKLTANLYTTYVTFKYRNVPIERQKLTL”), and 95-118 (“SQVFKYTGFIRTLAPGEDGADRAS”). The epitopes of the two proteins are almost identical, which indicated that the insertion of CTLA-4IgV at the N-terminal of EgG1Y162 antigen did not change the epitope distribution of EgG1Y162 antigen, and the EgG1Y162 antigen is able to perform its original function.

Many immune cells and molecules are involved after the host is stimulated by an antigen, among which antigen-presenting cells play an important role in immune cells' recognition of foreign antigens (28). Enhancing the antigen recognition and presentation abilities of antigen-presenting cells will inevitably lead to a stronger and more effective immune response (29). DCs are the most functional antigen-presenting cells in the body (30,31). B7, also named CD80, expressed on the membrane of antigen-presenting cells binds to CD28/CTLA-4 expressed on the membrane of T cells, which regulate the activation of T cells (32). When the recombinant protein was docked with B7 using HDOCK software, we found that the docking between CTLA-4IgV-EgG1Y162 and B7 was better than those between EgG1Y162 and B7. The formed complex was more stable and had more amino acid residues than the binding sites. In order to verify the docking results predicted by bioinformatic analysis, purified His-EgG1Y162 and His-CTLA-4IgV-EgG1Y162 recombinant proteins were used as antigens to bind to DCs *in vitro*. The results showed that compared with HIS-EgG1Y162, DCs had a stronger binding ability to HIS-CTLA-4IgV-EgG1Y162 and were more likely to mature under stimulation. Further, the results of the *in vivo* experiment clearly showed that HIS-CTLA-4IgV-EgG1Y162 promoted the maturation of DC in spleen and induced a stronger humoral immunity compared to HIS-EgG1Y162, which was consistent with the results of the *in vitro* experiment. These results also suggested that CTLA-4IgV could promote the detectability of DC for the antigen EgG1Y162 and enhance the immune response by promoting DC maturation.

The proliferation of lymphocytes increased the situation of the immune response in the body (33). Compared to the EgG1Y162 group, the proliferation of lymphocytes in the spleen of mice stimulated by CTLA-4IgV-EgG1Y162 increased significantly (P<0.05). It suggested that CTLA-4IgV-EgG1Y162 could bind to the antigen-presenting cells efficiently and accurately, thus enhancing the ability of antigen presentation and improving the efficiency of activated T cells.

Th1 cells secret IFN-γ and IL-2, which promote the activation and proliferation of CTL, NK, and macrophage. It finally induces cytotoxicity and plays an important role in anti-parasite infection (34). Th2 cells secret IL-4 and IL-10, which help the proliferation of B lymphocytes and the production of antibody (35). Th1 and Th2 have mutual adjustment, conferring a state of dynamic balance (36). We observed the immune response in mice injected by the vaccine through detecting the level of IFN-γ and IL-4 in serum. The results showed that the level of IFN-γ and IL-4 in the serum of mice immunized with recombinant protein CTLA-4IgV-EgG1Y162 were significantly higher than those in the serum of mice immunized with recombinant protein EgG1Y162 (P<0.05). Therefore, compared to EgG1Y162, the immune response of mice injected with CTLA-4IgV-EgG1Y162 was stronger.

The effect of the vaccine could be reflected by the level of antibody produced by the vaccine. We detected the titers of antibody against EgG1Y162 protein in the serum of each group of mice. The titers of IgG in mice immunized by CTLA-4IgV-EgG1Y162 was 1:24,300, which was more than those in the mice immunized by EgG1Y162 (P<0.05). At the same time, the immune response induced by recombinant proteins CTLA-4IgV-EgG1Y162 or EgG1Y162 was mainly due to Th2-type humoral immune response. The IgV region of CTLA-4 is very effective in enhancing the immune response of DNA vaccine, particularly Th2 response and CTL response activities after the animals were immunized with a DNA vaccine conjugated to CTLA-4IgV (37). Our experiment also proved that the immune response was greatly improved when the body was immunized by CTLA-4IgV-EgG1Y162.

In summary, we successfully constructed the recombinant protein CTLA-4IgV-EgG1Y162. The insertion of the N-terminal CTLA-4IgV promoted the binding of EgG1Y162 to DC and the maturation of DC. Preliminary exploration of the immune effect of CTLA-4IGV-Egg1Y162 showed that the insertion of CTLA-4IgV indeed enhanced its immune effect. However, the immune effect and specificity of the recombinant protein CTLA-4IgV-EgG1Y162 in intermediate hosts still need further experimental verification.

## Supplementary Material

Click to view [pdf].
