# Supplementary material for: Bioinformatic features and immunological response of recombinant antigen CTLA4-IgV-EgG1Y162 against Echinococcus granulosus
Source: Braz J Med Biol Res. 2024 Nov 25;57:e13139. doi: 10.1590/1414-431X2024e13139 (PMC11653501; doi:10.1590/1414-431X2024e13139)
Supplement: Supplementary file 1 [file 1414-431X-bjmbr-57-e13139-suppl.pdf]

Figure S1. EgG1Y162 protein. The red flag indicates a high prediction score.

Protein sequence used for prediction:

```
VDPELMAKLTKELKTTLPEHFRWIHVGSRSLELGWNATGLANLHADHIKL
TANLYTTYVTFKYRNVPIERQKLTLEGLKPSTFYEVVVQAFKGGSQVFKY
TGFIRTLAPGEDGADRASGF
```

Epitope predicted inside protein below:

Rank	Location	Epitope	Score	Recommend*
1	19 - 38	EHFRWIHVGSRSLELGWNAT	1.000	🚩
2	60 - 79	TFKYRNVPIERQKLTLEGLK	0.820	

Figure S2. CTLA-4IgV–EgG1Y162 protein. The red flag indicates a high prediction score.

Protein sequence used for prediction:

```
NVTQPPVVLASSRGVASFTCEYESSGKADEVRVTVLRKAGIQVTEVCAGT
YMVEDELTFLDDSSCIGTSRGNKVNLTIQGLRAMDTGLYVCKVELMYPPP
YYMGEGNGTQIYVIDPEPCPDSDGTDDDDKAMADIGSEFVDPELMAKLTK
ELKTTLPEHFRWIHVGSRSLELGWNATGLANLHADHIKLTANLYTTYVTF
KYRNVPIERQKLTLEGLKPSTFYEVVVQAFKGGSQVFKYTGFIRTLAPGE
DGADRASGF
```

Epitope predicted inside protein below:

Rank	Location	Epitope	Score	Recommend*
1	127 - 146	DDDKAMADIGSEFVDPELMA	1.000	🚩
2	158 - 177	EHFRWIHVGSRSLELGWNAT	0.947	🚩
3	199 - 218	TFKYRNVPIERQKLTLEGLK	0.776	
4	16 - 35	ASFTCEYESSGKADEVRVTV	0.649	
5	42 - 61	QVTEVCAGTYMVEDELTFLD	0.586	
6	75 - 94	NLTIQGLRAMDTGLYVCKVE	0.510	

Table S1. T/B cell antigen epitope prediction of recombinant proteins.

Recombinant proteins	Position		T/B combined epitope
	B-cell antigen epitope	T-cell antigen epitope	
EgG1Y162	19–28	7–18	***AKLTKELKTTLP***EHFRWIHVGS
	60–75	47–71	***HIKLTANLYTTYVTFKYRNVPIERQ***KLTL
	108–117	95–118	***SQVFKYTGFIRTLAPGEDGADRAS***
CTLA-4IgV–EgG1Y162	158–167	146–160	***AKLTKELKTTLPEHF***RWIHVGS
	199–209	184–208	***ADHIKLTANLYTTYVTFKYRNVPIE***R
	247–256	234–250	***SQVFKYTGFIRTLAPGE***DGADRA

Bold italic type indicates the T epitope and the B epitope is in the box.

Table S2. Analysis of the maturation of dendritic cells (DCs) in the spleen of mice in each group.

Groups	Maturation of DC (%)
Control	11.720 ± 1.539
Negative	9.436 ± 1.472
CTLA-4IgV–EgG1Y162	16.360 ± 2.414*#
EgG1Y162	11.010 ± 2.299

Data are reported as means and SD (n=6). *P<0.05 compared to control group and negative group; #P<0.05 compared to EgG1Y162 group (ANOVA).

Table S3. CCK8 results of the proliferation of splenic lymphocytes in each group.

Groups	OD value
Control	0.333 ± 0.024
Negative	0.331 ± 0.021
CTLA-4IgV–EgG1Y162	0.456 ± 0.012*#
EgG1Y162	0.390 ± 0.023*

Data are reported as means and SD (n=6). *P<0.05 compared to control group and negative group; #P<0.05 compared to EgG1Y162 group (ANOVA).

Table S4. Expression levels of cytokines interleukin (IL)-4 and interferon (IFN)-γ in peripheral blood of mice detected by ELISA.

Groups	IL-4 (pg/mL)	IFN-γ (pg/mL)
Control	6.801 ± 1.726	14.794 ± 4.777
Negative	7.154 ± 3.483	16.520 ± 4.451
CTLA-4IgV–EgG1Y162	15.669 ± 3.346*#	31.768 ± 4.409*#
EgG1Y162	11.611 ± 1.432*	24.662 ± 3.117*

Data are reported as means and SD (n=6). *P<0.05 compared to control group and negative group; #P<0.05 compared to EgG1Y162 group (ANOVA).
